# Aetiology and haemodynamic patterns of orthostatic hypotension in a tertiary syncope unit

**DOI:** 10.1093/europace/euaf017

**Published:** 2025-01-17

**Authors:** Madeleine Johansson, Boriana S Gagaouzova, Ineke A van Rossum, Roland D Thijs, Viktor Hamrefors, J Gert van Dijk, Artur Fedorowski

**Affiliations:** Department of Clinical Sciences, Lund University, Jan Waldenströms gata 35, 214 28 Malmö, Sweden; Department of Cardiology, Skåne University Hospital, 214 28 Malmö, Sweden; Department of Neurology and Clinical Neurophysiology, Leiden University Medical Centre, PO Box 9600, 2300 RC Leiden, Netherlands; Polikliniek Stichting Epilepsie Instellingen Nederland (SEIN), 2103 SW Heemstede, Netherlands; Department of Neurology and Clinical Neurophysiology, Leiden University Medical Centre, PO Box 9600, 2300 RC Leiden, Netherlands; Department of Neurology and Clinical Neurophysiology, Leiden University Medical Centre, PO Box 9600, 2300 RC Leiden, Netherlands; Polikliniek Stichting Epilepsie Instellingen Nederland (SEIN), 2103 SW Heemstede, Netherlands; Department of Clinical Sciences, Lund University, Jan Waldenströms gata 35, 214 28 Malmö, Sweden; Department of Cardiology, Skåne University Hospital, 214 28 Malmö, Sweden; Department of Neurology and Clinical Neurophysiology, Leiden University Medical Centre, PO Box 9600, 2300 RC Leiden, Netherlands; Department of Clinical Sciences, Lund University, Jan Waldenströms gata 35, 214 28 Malmö, Sweden; Department of Cardiology, Karolinska University Hospital, Eugeniavägen 3, 171 64 Solna, Stockholm, Sweden; Department of Medicine, Karolinska Institute, 171 64 Solna, Stockholm, Sweden

**Keywords:** Haemodynamics, Orthostatic hypotension, Pathophysiology, Tilt table test

## Abstract

**Aims:**

Orthostatic hypotension (OH) is an important differential diagnosis in unexplained syncope. Neurogenic OH (nOH) has been postulated to differ from non-neurogenic OH (non-nOH), yet pathophysiological differences are largely unexplored. We aimed to investigate aetiology and tilt table test (TTT)-induced haemodynamic responses in symptomatic OH patients.

**Methods and results:**

We performed a retrospective study analysing patients referred for unexplained syncope or highly symptomatic orthostatic intolerance with TTT-verified classical OH (cOH). Medical records were analysed for the presumptive aetiology of cOH. Fifty-two patients (mean age 73 ± 9 years, 46% women) with good quality TTT recordings were divided into three groups on clinical grounds: nOH, non-nOH, and mixed OH. The log-ratio (LR) method was applied to compare the decrease in mean arterial pressure (MAP_LR_) and corresponding contributions of heart rate (HR_LR_), stroke volume (SV_LR_), and total peripheral resistance (TPR_LR_) during the upright phase of TTT. The prevalence of cOH was 12 (23%) nOH, 14 (27%) non-nOH, and 26 (50%) mixed OH. No difference in MAP_LR_ was observed among the three groups during the 4th upright minute of TTT (nOH: −0.10 ± 0.04 vs. non-nOH: −0.07 ± 0.05 and vs. mixed OH: −0.06 ± 0.05, *P* = 0.10). The contributions of HR_LR_, SV_LR_, and TPR_LR_ to the drop in MAP_LR_ did not differ between groups (all *P* > 0.05).

**Conclusion:**

One-half of highly symptomatic OH patients had mixed OH, whereas one-quarter had either pure neurogenic or non-neurogenic OH, respectively. Different forms of OH were indifferentiable based on haemodynamic responses during TTT, questioning the clinical utility of such classification. Larger studies are needed to confirm these findings.

## Graphical Abstract

**Graphical Abstract:**
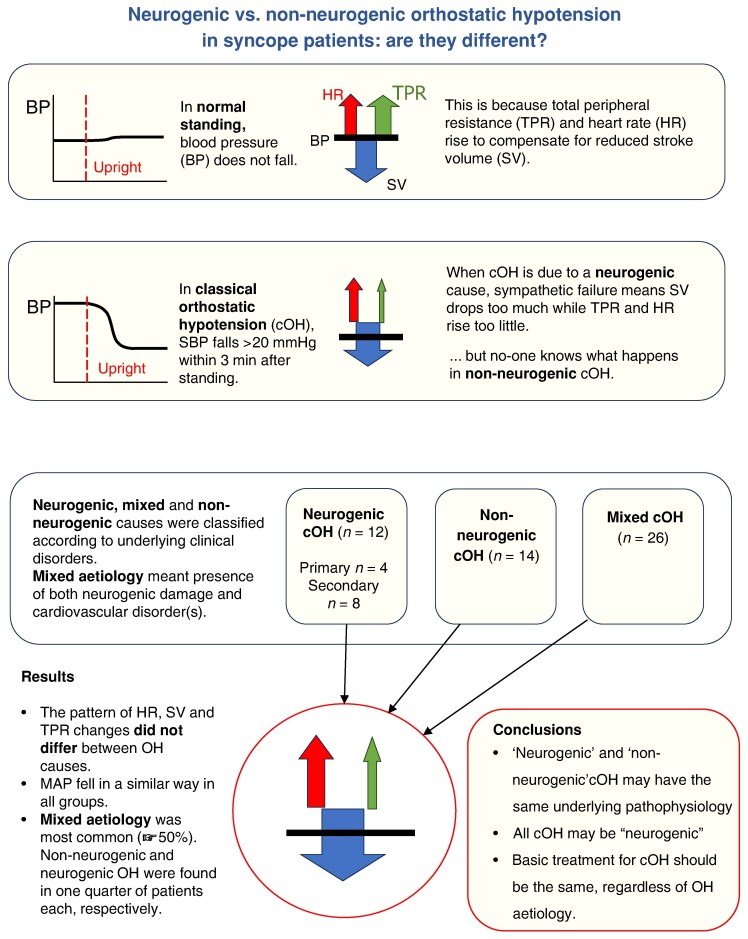

**What’s new?**

- Detailed haemodynamic differences comparing patients with neurogenic and non-neurogenic causes of orthostatic hypotension are largely unexplored.
- Our study illustrates that contributions of heart rate, stroke volume, and total peripheral resistance to low blood pressure did not differ between neurogenic and non-neurogenic orthostatic hypotension, which raises doubts about the clinical value of OH dichotomization into neurogenic vs. non-neurogenic form.
- In a specialized syncope unit, one-half of symptomatic OH patients had a mixed aetiology (both neurogenic and non-neurogenic) of OH, whereas pure neurogenic OH was seen in only one-fourth.

## Introduction

Classical orthostatic hypotension (OH) is defined as a sustained decrease in systolic BP (SBP) ≥ 20 mmHg and/or diastolic BP (DBP) ≥ 10 mmHg within 30 s to 3 min of active standing or tilt table test (TTT).^1,2^ Classical OH is associated with increased morbidity and mortality and leads to a significant number of hospital admissions.^3,4^

The aetiology of classical OH is multifactorial and traditionally divided into neurogenic OH (nOH), non-neurogenic (non-nOH), or mixed aetiology.^5,6^ Neurogenic causes include both primary neurodegenerative disorders caused by abnormal accumulation of α-synuclein in the nervous system (Parkinson’s disease, Lewy bodies dementia, multiple system atrophy, and pure autonomic failure), and secondary neurogenic causes, for example metabolic (diabetes mellitus), peripheral neuropathy, spinal cord problems, autoimmune, endocrine, and renal diseases etc.^7,8^ Non-neurogenic causes include medication, volume depletion, anaemia, and cardiovascular disease (e.g. heart failure, chronotropic incompetence, hypertension, and valvular heart diseases).^6,9^ Additionally, there are patients who exhibit a combination of both neurogenic and non-neurogenic causes of OH, recently proposed to be labelled ‘mixed aetiology’.^6^

It has been claimed that differences in aetiology correlate with different haemodynamic profiles. Patients with nOH are thought to exhibit little or no increase in HR in the upright position, whereas patients with non-OH have marked tachycardia.^7^ Further, nOH is often assumed to depend on peripheral vasoconstriction failure, i.e. an inability of total peripheral resistance (TPR) to increase sufficiently in response to a downward blood volume shift. However, detailed haemodynamic patterns comparing patients with nOH vs. non-nOH have been largely unexplored.^10,11^

In this study, we aimed to analyse the haemodynamic components contributing to nOH and non-nOH, respectively, and to quantify the contributions of HR, stroke volume (SV), and TPR to BP changes, using the log-ratio (LR) method.^12^ We decided to classify patients with classical OH based on clinical findings with a direct impact on the aetiology of OH, including symptoms and signs of neurological conditions that can present with OH, comorbidities, and medication use. We used exclusively clinical features for OH classification to prevent circular reasoning as haemodynamic variables may function both as inclusion and as outcome parameters.

## Methods

### Study population and design

The Syncope Study of Unselected Population in Malmö (SYSTEMA) cohort enrolled 3081 patients between 2008 and 2021 with unexplained syncope and/or pronounced symptoms of orthostatic intolerance that were referred to the tertiary syncope unit at Skåne University Hospital in Malmö to undergo TTT.^13–15^ Additional tests were performed whenever indicated, e.g. exercise test, ambulatory prolonged electrocardiogram (Holter ECG), 2D transthoracic echocardiography, coronary and pulmonary angiography, brain imaging, and electroencephalography.^15,16^

**Figure 1:**
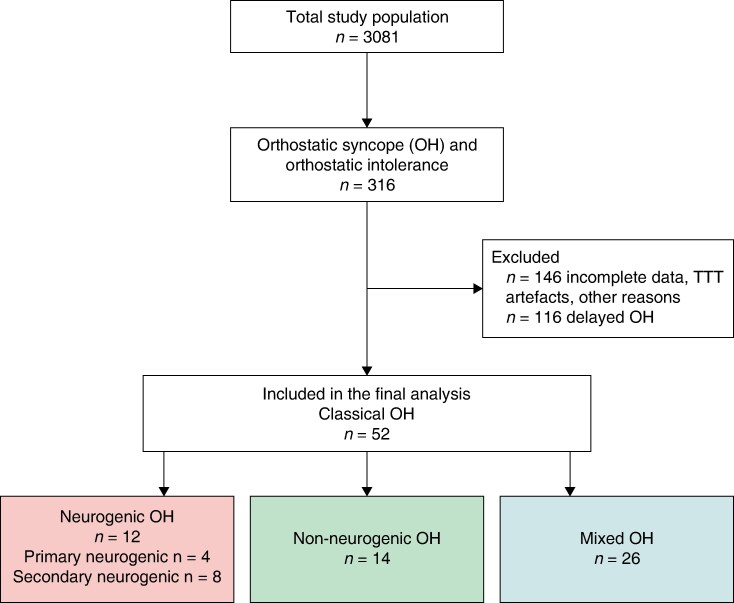
Flowchart. OH, orthostatic hypotension; POTS, postural orthostatic tachycardia syndrome; TTT, tilt table test; VVS, vasovagal syncope.

The flowchart is illustrated in *Figure 1*. Of 3081 patients, 316 were diagnosed with classical OH or delayed OH verified by TTT according to current syncope and TTT guidelines^1,17^; initial OH was excluded.^1,18^ Of these, 170 met diagnostic criteria of classical OH, the rest being delayed OH.^19^ After a detailed analysis of medical records and TTT recordings, 52 patients were selected based on inclusion criteria, i.e. TTT-proven classical OH, age ≥ 18 years, absence of other TTT diagnoses such as delayed OH, vasovagal syncope, or carotid sinus syndrome, good quality of TTT data for haemodynamic interpretation, and complete clinical data allowing to classify OH aetiology as neurogenic, non-neurogenic, or combined (mixed).

### Classifications of prevalent and incident neurogenic and non-neurogenic orthostatic hypotension

We analysed available medical records retrospectively. The mean time period screened was 4 years (range 0–9 years) before and after TTT, to identify the underlying cause of OH prior to undergoing TTT, i.e. prevalent neurogenic/non-neurogenic causes. We divided patients into three groups: nOH, non-OH, and mixed OH.^6,9,20,21^

- nOH was defined as being diagnosed with a *primary neurogenic* cause of OH (Parkinson’s disease, Lewy body dementia, multiple system atrophy, and pure autonomic failure). *Secondary neurogenic* cause of OH included the presence of one or more of following diseases: peripheral neuropathy [e.g. diabetes mellitus, chronic kidney disease (stage ≥ 3), vitamin B12/folate deficiency, amyloidosis, autoimmune disease (e.g. rheumatic disease, systemic lupus erythematosus, multiple sclerosis, and autoimmune thyroid diseases)], spinal cord injury (e.g. spinal cord compression and spinal cord tumours), cerebral tumours, and dementia due to other causes than Lewy body (e.g. Alzheimer’s disease or vascular dementia).^20–22^ There had to be no clues for a disorder in the list of non-neurogenic causes mentioned below.
- Non-nOH was defined as the absence of any neurogenic cause mentioned above and the presence of a positive non-neurogenic cause of OH, including medications having a negative impact on the cardiovascular system, such as intake of nitrates, antihypertensives, diuretics, tricyclic antidepressants, and chemotherapeutics; cardiac causes included heart failure, arrhythmias, in particular chronotropic incompetence, valvular pathology including aortic stenosis and mitral regurgitation, pulmonary hypertension, systemic hypertension, history of myocardial infarction, and myocarditis with an impact on ventricular function; finally, hypovolemic causes included anaemia, dehydration, or venous pooling.^6,20^
- Mixed OH was defined as the presence of both secondary neurogenic and non-neurogenic causes prior to undergoing TTT. In unresolved cases, the investigators reviewed the medical charts post-test to determine whether a concealed neurogenic pathology was revealed during follow-up period.

All classifications were based on a consensus discussion (M.J./A.F.) and based solely on prevalent diseases identified in the medical records, blinded to the haemodynamic responses observed during TTT.^23^ Patients who could not be allocated to one of the three groups were excluded from analysis but counted, as demonstrated in *Figure 1*. Detailed neurogenic and non-neurogenic diagnoses are provided in *Table 1*.

**Table 1:** Characteristics of the study population (*n* = 52)

	nOH (*n* = 12)		Mixed OH (*n* = 26)	Non-nOH (*n* = 14)	*P*-value
	Primary nOH (*n* = 4)	Secondary nOH (*n* = 8)			
Age, years ± SD (range)	64.8 ± 10.1 (50–73)	68.4 ± 8.7 (55–84)	75.0 ± 8.5 (55–92)	74.7 ± 10.2 (54–94)	0.08
Sex, women, *n* (%)	3 (75.0)	3 (37.5)	13 (50.0)	5 (35.7)	0.50
BMI, kg/m^2^ ± SD	23.6 ± 4.7	25.0 ± 5.0	26.4 ± 4.9	27.3 ± 2.7	0.42
Plasma adrenaline, nmol/L^a^					
Supine	0.14^b^	N/A	0.18 ± 0.13	0.21 ± 0.22	0.92
Upright 3 min TTT	0.24^b^		0.46 ± 0.39	0.35 ± 0.34	0.84
Plasma noradrenaline, nmol/L^a^					
Supine	1.5^b^	N/A	3.07 ± 0.49	2.31 ± 0.80	0.2
Upright 3 min TTT	1.9^b^		4.46 ± 0.23	4.07 ± 2.18	0.52
Prevalent primary neurogenic diseases, *n* (%)					
Multiple system atrophy	2 (50.0)	0	0	N/A	N/A
Parkinson’s disease	1 (25.0)	0	0		
Lewy body dementia	1 (25.0)	0	0		
Pure autonomic failure	0	0	0		
Prevalent secondary neurogenic diseases, *n* (%)					
Cerebral tumour	0	0	1 (3.8)	N/A	N/A
Dementia (other than Lewy body)	0	0	2 (7.7)		
Spinal cord injury	0	5 (62.5)	9 (34.6)		
Peripheral neuropathy					
Diabetes mellitus	0	5 (62.5)	3 (11.5)		
Chronic kidney disease	0	4 (50.0)	6 (23.1)		
Vitamin B12/folate deficiency	0	0	1 (3.8)		
Autoimmune diseases	0	3 (37.5)	10 (38.5)		
Prevalent non-neurogenic diseases, *n* (%)					
Heart failure	N/A		7 (26.9)	0	N/A
Coronary artery disease			3 (11.5)	3 (21.4)	
Myocardial infarction			2 (7.7)	3 (21.4)	
Hypertension			17 (65.4)	8 (57.1)	
Atrial fibrillation/flutter			8 (30.8)	2 (14.3)	
Bradyarrhythmias			5 (19.2)	4 (28.6)	
Myocarditis			0	1 (7.1)	
Aortic stenosis			2 (7.7)	1 (7.1)	
Cancer of non-neurogenic origin			4 (15.4)	4 (28.6)	
Hypovolaemia					
Dehydration			0	1 (7.1)	
Chronic diarrhoea			0	1 (7.1)	
Medications					
Antihypertensives			17 (65.4)	8 (57.1)	
Diuretics			26 (100.0)	14 (100.0)	
Vasodilators			3 (11.5)	3 (21.4)	
Chemotherapeutic agents			4 (15.4)	4 (28.6)	
Antidepressants			5 (19.2)	2 (14.3)	

BMI, body mass index; nOH, neurogenic orthostatic hypotension; non-nOH, non-neurogenic orthostatic hypotension; OH, orthostatic hypotension; TIA, transient ischaemic attack; TTT, tilt table test.

^a^Data only available in a subset of *n* = 10 patients.

^b^Data only available in one patient.

### Tilt table test protocol

Patients were taking their regular medications and instructed not to eat for 2 h prior to examination but were allowed to drink water at will. They were asked to fill out a questionnaire about past medical history. Patients were placed on a tilt table and rested for at least 10 min to obtain haemodynamically stable parameters; thereafter a standardized 70° TTT was carried out for 20 min according to current recommendations.^17,20^ As all classical OH patients demonstrated a profound and diagnostic BP fall during passive TTT phase, nitroglycerine provocation was omitted. Beat-to-beat BP and ECG were monitored continuously by a validated non-invasive photoplethysmographic method with a wrist unit and finger cuff of appropriate size (Nexfin monitor; BMEYE, Amsterdam, Netherlands, or Finapres Nova, Finapres Medical Systems, Enschede, Netherlands).^24,25^

### Haemodynamic analysis

The relationship between BP, HR, SV, and TPR can be expressed by the following equation: cardiac output (CO) is mean arterial pressure (MAP) divided by TPR (CO = MAP/TPR). As CO is the product of HR and SV, the equation can be expressed as: MAP = HR·SV·TPR.

Beat-to-beat data of the entire TTT were obtained, along with remarks indicating times of tilt up and tilt down. Systolic BP, MAP, DBP, HR, SV, and TPR data were read from these files and linearly interpolated to obtain regularly spaced data at one sample per second. We inspected the resulting files, and removed artefacts (e.g. movement, occasional extrasystolic beats, and calibration) as well as additional provocative measures, such as carotid sinus massage. Data periods were deleted if such periods lasted a short time and were preceded and followed by stable data with similar mean values, they were linearly interpolated instead. Files containing frequent extrasystolic beats or apparent atrial fibrillation were deleted. After data cleaning, the mean of a supine baseline period lasting 60 s before TTT (from −180 to −20 s) was calculated for each patient for SBP, MAP, DBP, HR, SV, and TPR.

For each of MAP, HR, SV, and TPR, the entire time series was then expressed as a ratio of the mean of the baseline period, which itself thereby had a value of one. The logarithm (base 10) was then taken to produce LRs, meaning that the baseline then had a value of zero and relations became additive: for each point in time, MAP_LR_ = HR_LR_ + SV_LR_ + TPR_LR_. Reactivity to TTT was characterized by means over the 4th minute after tilt, in view of the common use that defines classical OH 3 min after TTT.^12^

### Ethical approval

The regional ethical review board in Lund, Sweden approved the study protocol (reference no. 82/2008). All study participants gave written informed consent. The study complied with the Declaration of Helsinki and its later amendments.

### Statistical analysis

The main characteristics of the study population are presented as mean and standard deviation for continuous variables, and as counts and percentages for categorical variables. Intergroup differences were analysed using ANOVA for non-categorical variables and Pearson’s χ^2^ test for categorical variables. For comparative purposes, BP differences were calculated by subtracting upright from supine values. We also compared plasma catecholamine levels (adrenaline and noradrenaline) collected during TTT in the supine position and after 3 min of TTT in a subset of 10 patients with available data. Matlab was used for statistical testing. *P*-values of <0.05 were considered significant; values 0.05 < *P* < 0.10 were regarded as trends.

## Results

Study characteristics are displayed in *Table 1*. Out of 52 patients (mean age 73.1 ± 9.5 years, 46% women) who fulfilled inclusion criteria, 83% (43/52) had a history of syncope, while the rest reported pronounced orthostatic intolerance and presyncope episodes. A detailed clinical record analysis of the probable cause of OH resulted in 12 (23%) patients classified as nOH, 14 (27%) as non-nOH, and 26 (50%) as having a mixed aetiology of classical OH. No significant differences were observed between the groups in terms of age, sex, and BMI (*P* > 0.05).

### Supine and tilt table test haemodynamics

Haemodynamic data in the supine position and during the 4th minute of TTT are presented in original measurement units in *Table 2*. We did not observe significant differences between the three groups for any of the parameters, and therefore, no *post hoc* test was performed. A trend towards significance was noted for supine SBP and MAP, both being lower in patients with mixed OH compared with patients with nOH and non-nOH. A similar trend was found for upright SBP.

**Table 2:** Haemodynamic data in the supine position and during the 4th minute of tilt table testing (*n* = 52)

	Total population (*n* = 52)	nOH (*n* = 12)	Non-nOH (*n* = 14)	Mixed OH (*n* = 26)	ANOVA *P*-value
Supine position					
SBP mmHg ± SD	152.1 ± 27.0	160.8 ± 21.7	163.2 ± 30.2	143.4 ± 26.0	0.07
MAP mmHg ± SD	104.7 ± 16.2	112.2 ± 18.6	108.6 ± 18.5	99.0 ± 12.2	0.08
DBP mmHg ± SD	78.1 ± 12.5	83.2 ± 16.7	78.6 ± 13.5	74.6 ± 8.4	0.18
HR bpm ± SD	68.4 ± 11.3	72.9 ± 14.0	67.4 ± 10.0	66.5 ± 10.7	0.41
SV mL ± SD	87.2 ± 29.3	97.3 ± 36.6	87.6 ± 26.8	81.5 ± 27.8	0.49
TPR mmHg·min/L ± SD	0.020 ± 0.0063	0.018 ± 0.0076	0.020 ± 0.0056	0.021 ± 0.0062	0.59
TTT 4th minute					
SBP mmHg ± SD	120.3 ± 20.7	119.0 ± 16.1	131.5 ± 25.4	116.7 ± 18.5	0.07
MAP mmHg ± SD	87.8 ± 14.3	88.6 ± 14.7	92.4 ± 16.7	85.8 ± 12.9	0.48
DBP mmHg ± SD	70.7 ± 12.8	71.6 ± 16.9	71.9 ± 12.5	69.6 ± 11.4	0.95
HR bpm ± SD	76.6 ± 12.4	79.9 ± 14.0	75.4 ± 8.6	74.5 ± 13.4	0.25
SV mL ± SD	67.7 ± 28.0	74.7 ± 36.5	74.7 ± 33.7	61.2 ± 20.0	0.38
TPR mmHg·min/L ± SD	0.019 ± 0.0063	0.017 ± 0.0049	0.019 ± 0.0064	0.021 ± 0.0067	0.14

DBP, diastolic blood pressure; MAP, mean arterial pressure; nOH, neurogenic orthostatic hypotension; non-nOH, non-neurogenic orthostatic hypotension; OH, orthostatic hypotension; SBP, systolic blood pressure; SV, stroke volume; TPR, total peripheral resistance; TTT, tilt table test.

### Haemodynamic responses to the upright position

The differences between supine and upright measurements (ΔSBP, ΔDBP, and ΔMAP) were used to reflect OH severity (*Table 3*). Systolic BP, DBP, and MAP decreased considerably in all three groups, as expected, with the largest decrease observed in patients with nOH, although the difference between groups was not significant. The largest MAP decrease occurred in MAP in patients with nOH, whereas patients with mixed OH had the smallest drop.

**Table 3:** Haemodynamic responses and log-ratio analysis during tilt table test of the study population (*n* = 52)

	Total population (*n* = 52)	nOH (*n* = 12)	Non-nOH (*n* = 14)	Mixed OH (*n* = 26)	ANOVA *P*-value
BP difference					
ΔSBP mmHg ± SD	31.8 ± 18.6	41.9 ± 15.9	31.6 ± 18.6	26.7 ± 18.3	0.12
ΔMAP mmHg ± SD	16.9 ± 12.4	23.7 ± 9.7	16.2 ± 13.0	13.2 ± 11.2	0.06
ΔDBP mmHg ± SD	7.4 ± 9.4	11.5 ± 6.8	6.7 ± 10.6	5.1 ± 8.1	0.14
Log-ratio 4th minute					
MAP_LR_ ± SD	−0.08 ± 0.05	−0.10 ± 0.04	−0.07 ± 0.05	−0.06 ± 0.05	0.10
HR_LR_ ± SD	0.05 ± 0.05	0.04 ± 0.05	0.05 ± 0.04	0.05 ± 0.05	0.36
SV_LR_ ± SD	−0.12 ± 0.08	−0.13 ± 0.08	−0.09 ± 0.09	−0.12 ± 0.08	0.38
TPR_LR_ ± SD	−0.03 ± 0.11	−0.04 ± 0.09	−0.06 ± 0.17	0.00 ± 0.09	0.44

BP difference denotes supine SBP minus upright SBP values.

BP, blood pressure; DBP, diastolic blood pressure; LR, log-ratio; MAP, mean arterial pressure; nOH, neurogenic orthostatic hypotension; non-nOH, non-neurogenic orthostatic hypotension; OH, orthostatic hypotension; SBP, systolic blood pressure; SV, stroke volume; TPR, total peripheral resistance; TTT, tilt table test.

A log-ratio analysis was performed to compare OH severity (MAP_LR_) and assess relative contributions to MAP_LR_ of HR_LR_, SV_LR_, and TPR_LR_ during TTT (*Table 3*). We did not observe any significant differences between the groups. Negative values of MAP indicate a decrease in MAP, accompanied by corresponding decreases in SV in all groups, whereas TPR either decreased, in patients with nOH and non-nOH, or did not change, in patients with mixed OH. HR increased in all three groups, without an intergroup difference.

**Figure 2:**
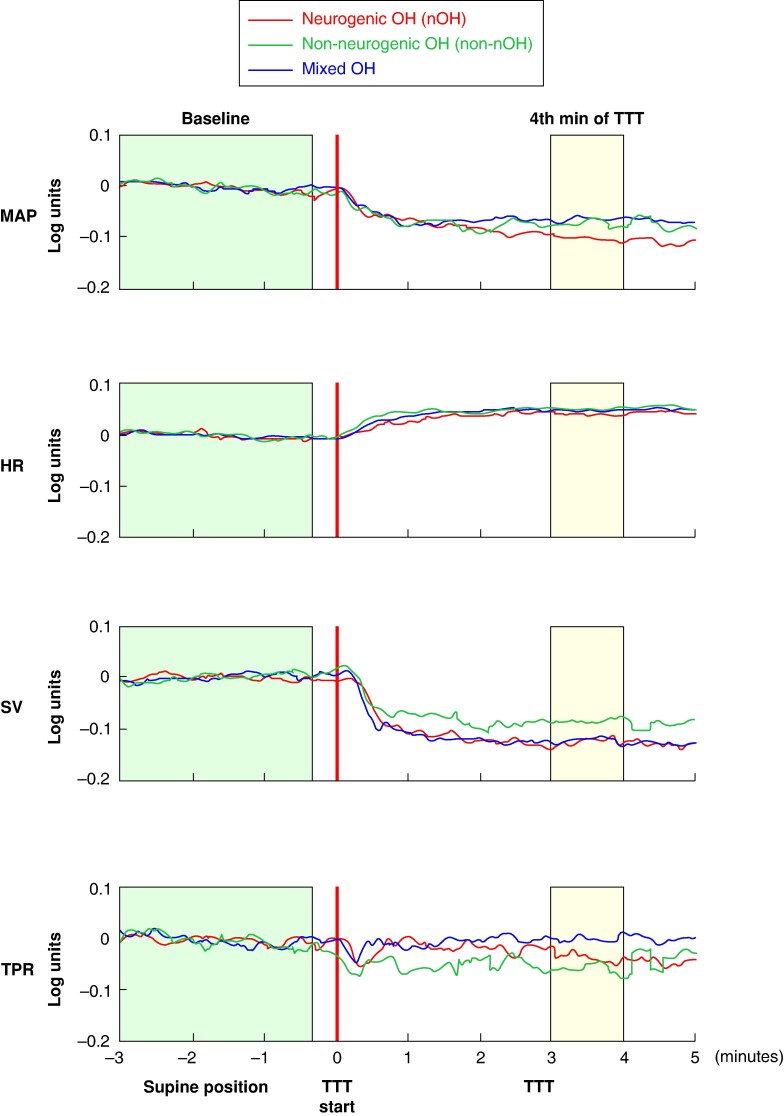
Log-ratio analysis of haemodynamic responses during tilt table testing in patients with classical orthostatic hypotension (*n* = 52). HR, heart rate; LR, log-ratio; MAP, mean arterial pressure; nOH, neurogenic orthostatic hypotension; non-nOH, non-neurogenic orthostatic hypotension; OH, orthostatic hypotension; SV, stroke volume; TPR, total peripheral resistance; TTT, tilt table test. The graph illustrates mean values of log-ratios of MAP_LR_, HR_LR_, SV_LR_, and TPR_LR_ on the *Y*-axis during different time periods of tilt table testing (*X*-axis) for patients with neurogenic (red), non-neurogenic (green), and mixed orthostatic hypotension (blue). The green and yellow boxes indicate the periods before TTT and 4 min after TTT, which were used to calculate supine and upright haemodynamic values for log-ratio analysis, respectively.

Fluctuations of MAP_LR_, HR_LR_, SV_LR_, and TPR_LR_ during TTT are shown in *Figure 2*, showing that the time course of HR_LR_, SV_LR_, and TPR_LR_ did not significantly differ between patients with nOH and non-nOH, indicating that the patterns of HR_LR_, SV_LR_, and TPR_LR_ did not differ between nOH and non-nOH.

## Discussion

We performed an aetiology-stratified analysis of haemodynamic changes provoked by TTT among patients diagnosed with classical OH. Our findings show no statistically significant differences in haemodynamic responses in patients with neurogenic OH compared with non-neurogenic OH and mixed aetiologies of classical OH. Moreover, in this symptomatic patient population, mixed aetiology (i.e. both neurogenic and non-neurogenic factors) was highly prevalent, occurring in one of two patients with OH. Neurogenic and non-neurogenic aetiologies were similarly frequent in the remaining group. Our data argue against the view that neurogenic OH constitutes a separate pathophysiological haemodynamic entity.

### Possible haemodynamic patterns in classical orthostatic hypotension

In the workup of unexplained syncope, especially in centres with access to more advanced autonomic tests, OH constitutes an important differential diagnosis, present in at least one of ten patients.^19^ Orthostatic hypotension depends largely on dysfunction of normal baroreflex response.^26^ Assuming the upright position results in venous pooling and plasma extravasation, which both reduce venous return to the heart, manifested as a decrease in SV, and consequently a decrease in cardiac output and blood pressure. The baroreflex responds to this drop in BP in two ways: (i) parasympathetic withdrawal and sympathetic activation resulting in an increase in HR and cardiac contractility, which can increase SV, and (ii) a sympathetic increase resulting in arteriolar vasoconstriction, which causes an increase in TPR (also known as systemic vascular resistance). In individuals with normal responses to orthostatic challenge, the increases in TPR and HR completely counteract the lowering of SV.^27^ All three mechanisms can theoretically contribute to classical OH: SV can become too low, and HR and TPR can increase too little.

We hypothesized that the mechanism by which these haemodynamic parameters influence OH might vary depending on the underlying aetiology. In sympathetic failure, which may be regarded the essence of nOH, the mechanism is well-known: a paresis of arteriolar vasoconstriction implies an inability to increase TPR sufficiently, suggesting that correction efforts must involve increases of HR or SV. For conditions resulting in non-nOH, the mechanisms have not been fully elucidated and may vary. We hypothesized that the circulating volume in hypovolaemia would be so low that SV becomes very low, requiring very large increases in HR or TPR. In patients with classical OH and bradyarrhythmias, we observed that low HR is a main factor to start with, and in patients with chronotropic incompetence the physiological increase in HR is impaired, leaving TPR as the main corrective influence.

### Differentiating neurogenic and non-neurogenic orthostatic hypotension

Although non-nOH is believed to be more prevalent than nOH,^28^ it has received much less pathophysiological attention than nOH.^6^ Neurogenic OH is currently diagnosed based on clinical history or by applying tests, such as the Valsalva test^29–32^ or the delta HR/SBP ratio.^10^ However, there appear to be no studies identifying non-nOH based on an independent gold standard. If sympathetic failure is indeed the hallmark of nOH,^10,21,33^ then estimates of TPR, available using devices measuring beat-to-beat continuous BP should be useful. Even so, estimates of TPR have not yet been proposed to distinguish between nOH and non-nOH. Instead, current tests rely on an indirect assessment of vasoconstriction failure, i.e. a failure of BP to increase attributable to vasoconstriction, observed in phases 2b and 4 of the Valsalva test, or through assessment of additional parasympathetic failure, i.e. a failure of HR to increase sufficiently during orthostasis. However, it is important to acknowledge that many pivotal studies on OH have been performed in centres with a special interest in OH due to neurodegenerative diseases such as Parkinson’s disease and multiple system atrophy, which may have introduced an unavoidable bias in patient selection. Even if healthy controls were added to these studies, lack of non-neurogenic OH and mixed aetiologies must have limited the generalizability of these data, which the current study aimed to overcome.

### Future perspectives

A recent viewpoint has suggested that classical OH is not a binary matter of neurogenic vs. non-neurogenic classical OH, but rather a spectrum of disease that always has a neurogenic component.^34^ Significant contributions have been made during the past decades in the field of syncope research enhancing our knowledge of the underlying mechanisms.^35,36^ At present, no studies have compared the importance of HR, SV, and TPR in patients with classical OH. A possible reason for this may be the difficulty in measuring and comparing all parameters. Moreover, only recently, the LR method to compare the influences of HR, SV, and TPR on MAP was developed.^12,37^ Prospective studies are needed to assess the competitive risk of death and syncopal recurrence in this patient population for a more detailed clinical characterization. Future studies should consider including a battery of tests of proven or suggested value to distinguish between nOH and non-nOH, thus allowing a comparison between clinical, haemodynamic, and ancillary test data.

### Strengths and limitations

An important strength of the current study is that the past medical history of each patient was carefully evaluated, independent of tilt table results, through a meticulous review of medical records and consensus discussion, thus limiting the incorrect diagnosis of nOH and non-nOH. To the best of our knowledge, this is the first paper diagnosing non-nOH on positive grounds. Further, all patients were evaluated by the same examination protocol in a centre with access to extensive diagnostic modalities and expert expertise. However, there are several important limitations of this study. The small study sample may have hampered identification of subtle statistical differences between nOH and non-nOH although it still argues against a fundamental qualitative difference between nOH and non-nOH. The cross-sectional design prevents any strong conclusions about the causal relationships between clinical history and OH aetiology, although we followed the same assessment protocol for each patient. As such, we lacked complete data on follow-up and syncopal recurrence. Also, the reproducibility of OH during TTT is limited, ∼70–80%, although the reproducibility increases when OH is accompanied by pathological autonomic function tests, which were not systematically performed in the studied cohort.^38^ Finally, the study was carried out in a tertiary centre for syncope and orthostatic intolerance, meaning that the findings may not represent those in the general population of OH, which are most often treated by a general practitioner.

In conclusion, our aetiology-stratified analysis indicated that there was no evidence of fundamental haemodynamic differences, provoked by a TTT, between patients with neurogenic, non-neurogenic, and mixed aetiologies of classical OH. Further larger independent cohort studies are warranted to verify these findings.

## Data Availability

The data supporting the findings of this study are available from the corresponding author on reasonable request.
